# Expanding the β-III Spectrin-Associated Phenotypes toward Non-Progressive Congenital Ataxias with Neurodegeneration

**DOI:** 10.3390/ijms22052505

**Published:** 2021-03-02

**Authors:** Paula Sancho, Amparo Andrés-Bordería, Nerea Gorría-Redondo, Katia Llano, Dolores Martínez-Rubio, María Eugenia Yoldi-Petri, Luba Blumkin, Pablo Rodríguez de la Fuente, Fernando Gil-Ortiz, Leonor Fernández-Murga, Ana Sánchez-Monteagudo, Vincenzo Lupo, Belén Pérez-Dueñas, Carmen Espinós, Sergio Aguilera-Albesa

**Affiliations:** 1 Unit of Rare Neurodegenerative Diseases, Centro de Investigación Príncipe Felipe (CIPF), 46012 Valencia, Spain; psancho91@hotmail.com (P.S.); dandres@cipf.es (A.A.-B.); mdmartinez@cipf.es (D.M.-R.); asanchez@cipf.es (A.S.-M.); vlupo@cipf.es (V.L.); 2 Department of Physiology, Faculty of Medicine and Dentistry, University of Valencia, 46010 Valencia, Spain; 3 Pediatric Neurology Unit, Department of Pediatrics, Complejo Hospitalario de Navarra, 31008 Pamplona, Spain; nerea.gorria.redondo@navarra.es (N.G.-R.); yoldi.petri@navarra.es (M.E.Y.-P.); 4 Clinical Psychology, Department of Psychiatry, Complejo Hospitalario de Navarra, 31008 Pamplona, Spain; katia.llano.ordonez@navarra.es; 5 Pediatric Neurology Unit, Wolfson Medical Center, Holon, Sackler School of Medicine, Tel-Aviv University, 69978 Tel-Aviv, Israel; luba.blumkin@gmail.com; 6 Pediatric Imaging Unit, Department of Radiology, Complejo Hospitalario de Navarra, 31008 Pamplona, Spain; pablo.rodriguez.delafuente@navarra.es; 7 CELLS-ALBA Synchrotron Light Source, 08290 Barcelona, Spain; fgil@cells.es; 8 Molecular Oncology Laboratory, Hospital Arnau de Vilanova, 46015 Valencia, Spain; malefermu67@gmail.com; 9 Pediatric Neurology Research Group, Vall d’Hebron Research Institute (VHIR), Universitat Autònoma de Barcelona, 08035 Barcelona, Spain; belen.perez@vhir.org; 10 Navarrabiomed-Fundación Miguel Servet, 31008 Pamplona, Spain

**Keywords:** *SPTBN2* gene, β-III spectrin, non-progressive congenital ataxia, neurodegeneration

## Abstract

(1) Background: A non-progressive congenital ataxia (NPCA) phenotype caused by β-III spectrin (*SPTBN2*) mutations has emerged, mimicking spinocerebellar ataxia, autosomal recessive type 14 (SCAR14). The pattern of inheritance, however, resembles that of autosomal dominant classical spinocerebellar ataxia type 5 (SCA5). (2) Methods: In-depth phenotyping of two boys studied by a customized gene panel. Candidate variants were sought by structural modeling and protein expression. An extensive review of the literature was conducted in order to better characterize the *SPTBN2*-associated NPCA. (3) Results: Patients exhibited an NPCA with hypotonia, developmental delay, cerebellar syndrome, and cognitive deficits. Both probands presented with progressive global cerebellar volume loss in consecutive cerebral magnetic resonance imaging studies, characterized by decreasing midsagittal vermis relative diameter measurements. Cortical hyperintensities were observed on fluid-attenuated inversion recovery (FLAIR) images, suggesting a neurodegenerative process. Each patient carried a novel de novo *SPTBN2* substitution: c.193A > G (p.K65E) or c.764A > G (p.D255G). Modeling and protein expression revealed that both mutations might be deleterious. (4) Conclusions: The reported findings contribute to a better understanding of the *SPTBN2*-associated phenotype. The mutations may preclude proper structural organization of the actin spectrin-based membrane skeleton, which, in turn, is responsible for the underlying disease mechanism.

## 1. Introduction

**Figure 1:**
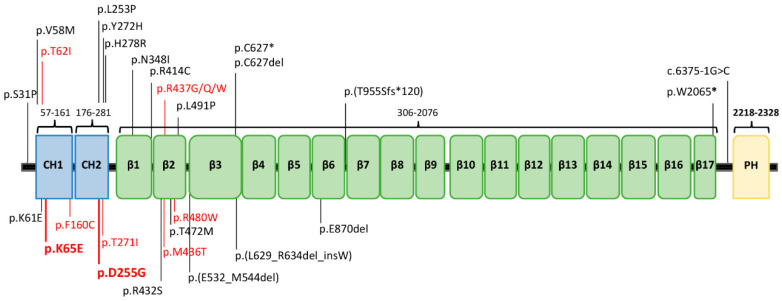
The protein β-III spectrin. (A) Protein structure (NP_008877.2) and location of its clinical mutations. Previously reported variants are in black, while the ones related to the non-progressive cerebellar ataxia (NPCA) phenotype are highlighted in red, including our two new mutations in bold font. CH1−CH2: calponin homology domains; β1−β17: β-spectrin domains; PH: pleckstrin homology domain.

Mutations in the gene encoding β-III spectrin (*SPTBN2*) lead to autosomal dominant (AD) spinocerebellar ataxia type 5 (SCA5), and spinocerebellar ataxia autosomal recessive type 14 (SCAR14) [1,2]. The SCA5 variant is more common than SCAR14 [3,4]. To date, 26 *SPTBN2* mutations, distributed along the entire coding region, have been linked to cerebellar ataxia (HGMD® Professional 2020.4; accessed on 19 February 2020) (Figure 1). β-III spectrin consists of three different regions: an N-ter actin-binding domain (ABD), which comprises the calponin homology (CH) domains; a central region containing 17 spectrin (β1-β17) repeat domains; and a C-ter pleckstrin homology (PH) domain.

Spectrins are crucial components of the cellular membrane skeleton, which support cell shape, maintain cell membrane integrity, and participate in cell adhesion and cell–cell contact [5]. They link the actin cytoskeleton to the cytoplasmic surface of the plasma membrane and participate in actin dynamics. β-III spectrin is primarily expressed in the cerebellum, where it is found in soma and dendrites of Purkinje cells, and has been linked to intracellular transport, Golgi apparatus (GA), and cytoplasmic vesicle dynamics [6,7,8]. Furthermore, β-III spectrin interacts with the glutamate transporter EAAT4 by stabilizing it at the plasma membrane [1,7,8].

As is usually the case with Mendelian disorders, AR inheritance is associated with more severe disease than AD inheritance. SCA5 is characterized by a slowly progressive pure cerebellar ataxia, and marked global cerebellar atrophy on magnetic resonance imaging (MRI) in adulthood, although early onset has also been reported [9,10,11,12]. Most patients may show ataxic gait, disabling action and postural tremor, pyramidal signs, dorsal column involvement, and gaze palsy. SCAR14 is an ataxia syndrome that may exhibit cerebellar atrophy on brain imaging, severe early-onset gait ataxia, eye movement abnormalities, developmental delay, and cognitive impairment [2,13,14]. An intermediate phenotype characterized by AD congenital cerebellar syndrome resembling SCAR14 was recently proposed. This variant has been associated with the p.R480W mutation [15].

We hereby report two unrelated cases of non-progressive congenital ataxia (NPCA), with progressive cerebellar volume loss on MRI and the appearance of cortical hyperintensities over time, in carriers of novel de novo *SPTBN2* mutations, c.764A > G (p.D255G) and c.193A > G (p.K65E). These new cases extend the β-III spectrin-associated phenotypes, and establish that heterozygous *SPTBN2* mutations can lead to NPCA with evidence of neurodegeneration on neuroimaging. Our hypothesis is supported by a comprehensive review of 11 cases that show a compatible SCAR14-like phenotype [4,11,12,15,16,17,18,19].

## 2. Results

### 2.1. Clinical, Neuroimaging, and Genetic Findings

Clinical, genetic, and neuroimaging features of the two unrelated patients are shown in Table 1. The patients fit the clinical diagnosis of NPCA, defined as congenital or early-onset ataxia without any progression or improvement on follow-up, after excluding prenatal, perinatal, and postnatal acquired disorders [20]. Both individuals were born after a normal pregnancy and had a normal perinatal history. Motor, language, and cognitive milestones improved with advancing age.

Full neuropsychological evaluations were completed in both cases (Figure S1). Two evaluations at 5 and 11 years of age in our first patient (MD-219; p.K65E mutation) demonstrated stable cognition in the range of mild–moderate intellectual disability (ID) (full IQ from 58 to 56), with a slight improvement of fluid reasoning (62 to 67), but worsening of processing speed over time (57 to 45). Remarkably, our first patient had good memory skills, especially in relation to social stories. General cognitive abilities at 8 years were borderline (full IQ 76) in our second patient (MD-207; p.D255G mutation), with median fluid reasoning (88), but low processing speed (56). Additionally, he fulfilled the criteria for attention deficit hyperactivity disorder (ADHD).

**Figure 2:**
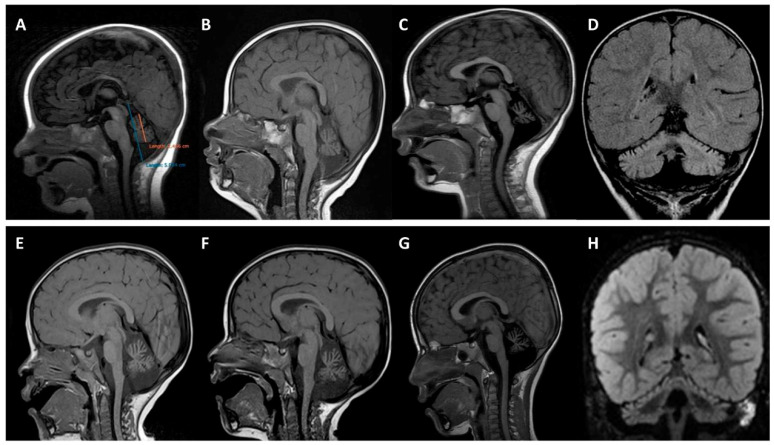
Brain magnetic resonance imaging. Upper panels (**A–D**) correspond to patient 1 (MD-219; p.K65E) and bottom panels (**E–H**) to patient 2 (MD-207; p.D255G). (**A**) Quantitative analysis using midsagittal vermis relative diameter (MVRD). The ratio (vermis diameter/total posterior cranial fossa diameter) was used to express the proportion of both values. The first image (**A**) corresponds to patient 1 at 4 months old; MVRD resulted in 54%. (**B–G**) Midline sagittal T1 demonstrating cerebellar vermian atrophy at the age of 12 (**B**) and 19 months (**C**) with an MVRD of 48% and 43% respectively, in patient 1; and at the age of 22 months (**E**), 29 months (**F**), and 6 years (**G**), with an MVRD of 59%, 57%, and 50%, respectively, in patient 2. (**D,H**) coronal fluid-attenuated inversion recovery (FLAIR) images exhibiting global cerebellar atrophy and predominantly superior cerebellar cortical hyperintensity (**D**) at 19 months old in patient 1, and (**H**) at 29 months old in patient 2.

Six MRIs from the two probands were analyzed (Figure 2). The first MRI of each patient, performed at 4 (patient 1, MD-219) and 12 (patient 2, MD-207) months of age, showed small cerebellar volumes with fissure enlargement. Subsequent MRIs revealed a progressive enlargement of the vermis and hemispheric inter-folia spaces. The atrophy was associated with FLAIR hyperintensities of the cerebellar cortex in both subjects, best observed on coronal sequences. Quantitative analysis of the cerebellum revealed a decreasing midsagittal vermis relative diameter (MVRD) over time.

The analysis of the panel MovDisord-498 revealed that both patients carried novel missense mutations in *SPTBN2* (NM_006946.3), c.193A > G (p.K65E), or c.764A > G (p.D255G), respectively (Table 1). We established that both genetic variants were de novo, since they were absent in the parents, supporting AD inheritance. In silico software (SIFT and Polyphen) predicted that the two mutations were probably damaging and deleterious. None of the variants were mentioned in any of the consulted databases (1000G, ESP6500, GnomAD, ClinVAR, and HGMD^®^ Professional 2019.4), and both mutations were classified as pathogenic according to the American College of Medical Genetics (ACMG) guidelines [21].

### 2.2. Modeling of the SPTBN2 p.K65E and p.D255G Mutations

The K65E and D255G variants are included in the CH1 and CH2 domains, respectively, of the human *SPTBN2* protein (NP_008877, SP_O15020) (Figure 1). To gain a structural understanding of the location of residues K65 and D255, a homology model, using as a template the available crystal structure of the ABD of plectin, was generated with Modeller [22]. The template shares 55% homology with the CH1–CH2 domains of the *SPTBN2* protein, covering the full sequence, with excellent alignment of the secondary structure elements, supporting the accuracy of the model.

**Figure 3:**
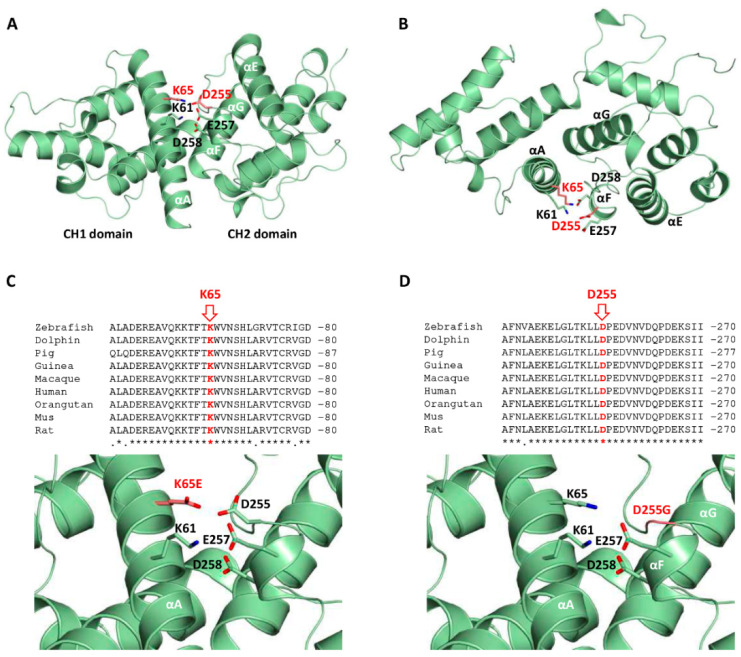
Structural homology model corresponding to the calponin homology (CH) domains of the human β-III spectrin and mutants. (**A,B**) Orthogonal views of the CH1 and CH2 domains (residues 47 to 291) highlighting the mutated residues K65 and D255 in red (in ball and stick representation). Surrounding residues and secondary structure elements are labeled. (**C,D**) Conservation of the mutated residues K65E and D255G, and close-up views of the mutants K65E and D255G (in red) and surrounding residues.

The mutated residues are located in helices A and F, each one belonging to a different CH domain. These charged residues in the native protein are well oriented and at a proper distance to establish a salt bridge, as occurs in the ABD of plectin (Figure 3a,b). Furthermore, these residues are close to several charged residues, including K61 (αA), E257, and D258 (αF), forming a surface salt bridge network contributing to the stabilization of the native structure (Figure 3a,b). The K65E mutation implies the replacement of the cationic and highly conserved residue by a positively charged residue, causing the disruption of the salt bridge with residue D255, and instability of the interface between the CH1 and CH2 domains (Figure 3c). The structure of helix A is probably preserved. Interestingly, the highly conserved residue D255 (Figure 3d) helps to orient lysines 61 and 65 (αA) belonging to the CH2 domain. As a result, the mutation to glycine would cause the loss of these electrostatic interactions, affecting the integrity of the surface salt bridge network. In addition, the introduction of a glycine residue at the N-ter end of helix F may importantly destabilize its secondary structure, causing deleterious effects. Altogether, mutation D255G may cause the perturbation of the interface between the CH1 and CH2 domains, and possibly their mutual three-dimensional arrangement.

### 2.3. Expression Analysis and Subcellular Location Studies

**Figure 4:**
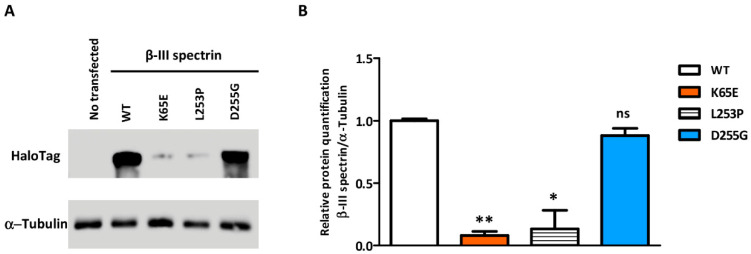
Western blot demonstrating the relative level of β-III spectrin in HeLa cells transfected with the different protein isoforms (WT or the p.K65E, p.L253P, and p.D255G mutants). (**A**) β-III spectrin immunodetected with an antibody against HaloTag, using tubulin as a load control. (**B**) Western blot-based quantification of β-III spectrin. Error bars: SEM; * *p* value < 0.05; ** *p* value < 0.01; ns: not significant.

The stability of overexpressed WT and mutated *SPTBN2* constructs transfected in HeLa cells was investigated by Western blot (WB) analysis. Compared to WT, D255G showed a reduced expression, whereas a notable reduction was observed for K65E as well as the pathological mutation L253P (Figure 4).

### 2.4. Phenotype to Genotype Correlations

A descriptive analysis of the known NPCA patients carrying heterozygous de novo *SPTBN2* mutations (including the two cases here described) is summarized in Table S1 [4,11,12,15,16,17,18,19]. All patients, including seven girls and six boys, presented with hypotonia and/or developmental delay before 12 months of age. All of them developed a cerebellar ataxia syndrome with a sluggish achievement of motor, cognitive, and language milestones during follow-up, which ranged from 2−18 years of age. All patients exhibited variable cognitive delays, ranging from intellectual disability (ID) to borderline or executive dysfunction, including: moderate ID in two (15%), mild in eight (62%), and borderline in three (23%). Seven patients (54%) were able to walk unsupported before age 5. Intention tremor was significant in more than half of the cases (54%), but normal ocular movements were described in only 15% (two patients). Few cases manifested additional clinical findings, such as bradykinesia (two), dystonia (one), or facial myokymia (one). Conversely, sequential cerebral MRI studies confirmed a progression of global cerebellar atrophy in spite of the clinical improvement in all cases. Cerebellar cortical hyperintensities, such as those seen in our patients, have not been reported.

The 13 patients exhibited a homogeneous NPCA phenotype with global cerebellar atrophy, and all of them harbored *SPTBN2* substitutions in heterozygosis. The 10 different missense variants fall in the calponin domains, CH1 domain (three mutations), CH2 domain (two mutations), and in the β2 domain (five mutations). Additional clinical signs, such as movement disorders or visual abnormalities, appear to have no correlation with the specific mutation and/or its location in the protein (Table S1).

## 3. Discussion

In this report, we describe two unrelated probands manifesting an NPCA phenotype, characterized by novel de novo *SPTBN2* mutations. Together with the 11 cases identified in our literature search, all 13 individuals represent a homogenous clinical syndrome consisting of infantile-onset hypotonia and/or developmental delay [4,11,12,15,16,17,18,19]. All patients developed a pure cerebellar syndrome with ataxic gait, dysmetria, slurred speech, abnormal ocular movements, and cognitive deficits [23]. At least seven probands had an intention tremor. Other movement disorders such as bradykinesia or dystonia are infrequent. Another consistent feature of this syndrome is the stability of the cerebellar syndrome, with slow improvement of motor, cognitive, and language development milestones over time.

Few reported patients had detailed neuropsychological evaluations, showing cognitive deficits ranging from normal–borderline IQ to moderate ID [4,16]. Our patient (MD-219) with the p.K65E mutation remained stable from a cognitive standpoint from 5 to 11 years of age, with some improvement of fluid reasoning over time. Our second patient’s cognitive ability was also better than verbal IQ, similar to patients carrying p.M436T or p.R437W [4,16].

This homogeneous non-progressive phenotype is paradoxically associated with a progression of cerebellar atrophy in serial neuroimaging during follow-up, despite preservation of other central nervous system structures. The cerebellar atrophy involves the vermis and hemispheres and can be related to diffuse cortical hyperintensity, mainly observed in our patients for the first time, on the upper part of hemispheres on FLAIR MRI sequences associated with the progression of the atrophy of cerebellar hemispheres. Interestingly, the cerebellar cortical hyperintensity pattern has been recognized in neurodegenerative disorders such as PLA2G6-associated neurodegeneration (PLAN) or congenital disorder of glycosylation type 1A (CDG1A) [24,25]. Other disorders with cerebellar involvement and regressive symptoms also present with cortical hyperintensities, including mitochondrial disorders, coenzyme Q deficiency, Christianson syndrome, and late-onset GM2 gangliosidosis [25]. However, this finding has also been reported in some patients with NPCA and pathogenic variants in *KIF1A*, *ITPR1, KCNC3, CACNA1A,* and *PMPCA* genes [26,27,28]. The cause of the bright cortex in the cerebellum is assumed to be a reactive gliosis to the neuronal cell loss and axonal swelling, as has been reported in CDG1A patients [29], but other mechanisms may be involved.

β-III spectrin^+/-^ mice show no sign of motor deficits or cerebellar degeneration, highlighting that haploinsufficiency is not enough to cause neurodegeneration [30,31], whereas the loss of β-III spectrin in *Mus musculus*, *Caenorhabditis elegans*, or *Drosophila melanogaster* causes axonal breakage [31,32]. Interestingly, for L253P, the normal axonal traffic is rescued at a lower temperature, suggesting a protein conformation defect [30], which may prevent the binding of the CH1 and CH2 domains to the actin cytoskeleton associated with the cytoplasmic side of the cell membrane [33]. The de novo mutations described in this report may destabilize the interface between the CH1 and CH2 domains, and additionally, D255G may affect the helical structure.

Protein expression studies revealed that K65E showed a notably reduced expression, similar to L253P [30], whereas D255G exhibited a slightly reduced expression. For many Mendelian disorders, mutations do not affect protein synthesis, but rather exert their effects by impairing protein folding or stability, which leads to rapid degradation [34]. Protein degradation occurs via two main pathways, the ubiquitin–proteasome system (UPS), and the autophagy–lysosome pathway, which are responsible for the clearance of aberrant peptides and play a neuroprotective role [35,36]. It is tempting to speculate that the unstable mutated proteins trigger a protein degradation pathway. However, despite the conformational defect with intracellular accumulation typical of L253P, the unfolded protein/endoplasmic stress response (UPR) did not appear to be induced [30].

In sum, we report two novel cases of *SPTBN2*-associated NPCA with cerebellar cortical hyperintensities, suggesting neurodegeneration even in the presence of a stable motor phenotype. Together with the 11 cases identified in the literature, the NPCA phenotype with cerebellar atrophy is being increasingly recognized beyond classical SCA5 and SCAR14 phenotypes. This homogeneous non-progressive phenotype is paradoxically associated with a progression of cerebellar atrophy. The pathomechanism may be associated with protein instability. Both mutations studied here may prevent the proper structural organization of the actin–spectrin-based membrane skeleton, whose perturbation within dendrites is supposed to underlie the disease mechanism [37,38].

## 4. Materials and Methods

### 4.1. Patients: Clinical and Neuroimaging Assessment

Within a multi-center cohort of 49 patients with early-onset cerebellar ataxia and/or cerebellar atrophy, two patients were identified with *SPTBN2* mutations and diagnosed with cerebellar ataxia, based on the validated scales scale for the assessment and rating of ataxia (SARA) [39,40,41] and international cooperative ataxia rating scale (ICARS) [42,43,44]. Clinical follow-up from the age at onset and MRI controls of both patients were done at the same hospital. Neuroimaging studies were performed on a 1.5T MR scan (Signa Excite, GE, Milwaukee, WI, USA) and included sagittal T1-weighted, axial T2-weighted, coronal FLAIR, diffusion, and T2*-/gradient-recalled echo images. Three pediatric neurologists and a pediatric neuroradiologist reviewed the MRI images. Cerebellar atrophy was qualitatively determined as a small cerebellar volume with widening of the foliae [45]. We achieved a quantitative analysis of the relative size of the vermis using the midsagittal vermis relative diameter (MVRD), calculated as the ratio of the vermis size over the total posterior cranial fossa size [24,46].

All protocols performed complied with the ethics guidelines according to the Declaration of Helsinki, and were approved by the Ethics Committee of the Hospital Universitari i Politècnic La Fe (Valencia, Spain; protocol code: 2019/0052 on 22 May 2019). The probands’ relatives signed an informed consent form for the study. We also performed a systematic review of patients diagnosed with NPCA as defined in [26], and heterozygous de novo *SPTBN2* mutations by searching MEDLINE through PubMed (accessed on 13 October 2020), using the following keywords: #1 *SPTBN2*, #2 non-progressive congenital ataxia, #3 congenital cerebellar ataxia, or #4 infantile-onset cerebellar ataxia. We selected eight articles that provided detailed clinical, neuroimaging, and genetic findings of 11 patients with NPCA and de novo *SPTBN2* mutations (Table S1), with the first case being reported in 2013 [4,11,12,15,16,17,18,19].

### 4.2. Genetics and Bioinformatics

Genetic analyses were performed using a customized panel with 498 genes involved in movement disorders (MovDisord-498) as previously described [47] and following filtering data criteria formerly reported in [48].

### 4.3. Conservation Analysis and Structural Modeling

Sequences of several organisms were downloaded from Ensembl (http://www.ensembl.org/; accessed on 20 June 2020), and multiple sequence alignment was performed using ClustalW2 (http://www.ebi.ac.uk/Tools/msa/clustalw2; accessed on 20 June 2020).

The structural homology model for amino acids 47 to 291 of the *SPTBN2* protein, corresponding to the CH domains, was generated by using residues 59 to 293 of the ABD (PDB 1MB8) as a template. The sequences were aligned with ClustalW2, followed by structural alignment and model building using Modeller [22]. The Z-score for the homology model is −1.39227. Structure superimposition of the β-III spectrin structural homology model to the template structure 1MB8 (plectrin) and mapping of the mutations K65E and D255G were performed in COOT v0.7 [49]. Figures were drawn using PyMOL (http://www.pymol.org/; accessed on 20 June 2020).

### 4.4. Protein Expression

The full length of the *SPTBN2* construct cloned in a pFN21A-HaloTag7 vector was acquired from Kazusa Genome Technologies (Promega, Madison, WI, USA). The *SPTBN2* missense mutations (K65E, L253P, or D255G) were introduced using a Site-Directed Mutagenesis Kit (Agilent, Santa Clara, CA, USA), and verified by sequencing. HeLa cells grown in DMEM supplemented with 10% heat-inactivated FBS, 5 g/L D-glucose, 1% P/S, and 1% L-glutamine were transfected for 24 h with 2 μg of cDNA per construct using FuGENE^®^ HD Transfection Reagent (Promega, Madison, WI, USA).

For Western blot (WB) analysis, 20 µg of protein were loaded on an 8% polyacrylamide gel under reducing conditions. Membranes were blocked with 5% milk powder in TBST (25 mm Tris-HCl, 150 mm NaCl, 0.05% Tween 20, pH 7.4) and incubated o/n with anti-HaloTag (Promega, Madison, WI, USA) and anti-α-tubulin (Santa Cruz Biotechnology, Dallas, TX, USA) in blocking buffer. The day after, membranes were incubated with the appropriate secondary HRP-conjugated antibodies (Invitrogen, Carlsbad, CA, USA) and revealed with Amersham™ Imager 600 (GE Healthcare, Chicago, IL, USA). WB was performed for triplicate and a Student’s *t*-test was used for statistical analysis.

## Figures and Tables

**Table 1:** Clinical, genetic, and neuroimaging features of our patients with *SPTBN2* mutations.

Patient	1 (MD-219)	2 (MD-207)
Gender, age (years)	M, 11	M, 8
*SPTBN2* variant	c.193A > G	c.764A > G
Position	chr11:66483417	chr11:66481110
Mutation type	Missense	Missense
Protein change	p.K65E	p.D255G
Protein domain	CH1	CH2
Age at onset	4 months	12 months
First clinical features	Hypotonia and transient upgaze deviation	Motor delay
Head control achieved	9 months	3 months
Sitting unsupported	24 months	9 months
Walking features	Ataxic gait, supported	Ataxic gait, unsupported
Speech	Impaired, understandable	Impaired, difficult to understand
Ocular anomalies	Strabismus, horizontal nystagmus, dysmetria	Strabismus
Hypotonia	+	+
Corticospinal signs	-	-
Tremor	Action (mild)	Action (moderate)
Dystonia	-	-
Bradykinesia	+ (mild)	-
Bulbar dysfunction	-	-
SARA total score ^1^	19/40	17/40
ICARS total score ^1^	38/100	26/100
Global IQ (z-score)	51 (z = −3)	76 (z = −1.6)
Behavioral problems	No	ADHD
Clinical course	Non-progressive	Non-progressive
Cerebellar atrophy	Progressive	Progressive
Cortical hyperintensity on FLAIR images	+	+
Additional MRI findings	-	-

^1^ Both clinical scales were rated by two pediatric neurologists (SAA, NG). ADHD: attention deficit hyperactivity disorder, CH1 and CH2: calponin homology domains 1 and 2, ICARS: international co-operative ataxia rating scale (range from 0—mild to 100—severe), IQ: intellectual quotient by Wechsler Scale (WISC-V) mean 100 (SD 15), SARA: scale for the assessment and rating of ataxia (range from 0—mild to 40—severe), FLAIR: fluid-attenuated inversion recovery, MRI: magnetic resonance imaging.

## Data Availability

The data that support the findings of this study are contained within the article or supplementary material.

## Supplementary Materials

The following are available online at https://www.mdpi.com/1422-0067/22/5/2505/s1.
